# The resilience of *Triatoma dimidiata*: An analysis of reinfestation in the Nicaraguan Chagas disease vector control program (2010–2016)

**DOI:** 10.1371/journal.pone.0202949

**Published:** 2018-08-23

**Authors:** Kota Yoshioka, Ezequiel Provedor, Jennifer Manne-Goehler

**Affiliations:** 1 School of Tropical Medicine and Global Health, Nagasaki University, Nagasaki, Japan; 2 Doctor of Public Health Program, Harvard T.H. Chan School of Public Health, Boston, MA, United States of America; 3 Facultad Regional Multidisciplinaria—Estelí, Universidad Nacional Autónoma de Nicaragua—Managua, Estelí, Nicaragua; 4 Department of Medicine, Beth Israel Deaconess Medical Center, Boston, MA, United States of America; 5 Department of Global Health and Population, Harvard T.H. Chan School of Public Health, Boston, MA, United States of America; Universidade Federal do Rio de Janeiro, BRAZIL

## Abstract

**Background:**

The control of *Triatoma dimidiata*, a major vector of Chagas disease, was believed to eliminate *Trypanosoma cruzi* transmission in Central America. This vector was known for its ability to repeatedly reinfest human dwellings even after initial insecticide spraying. Current vector control programs assume that community-based surveillance can maintain low levels of infestation over many years, despite a lack of evidence in the literature to corroborate this assumption. This study aims to evaluate long-term reinfestation risk in the Nicaraguan vector control program from 2010 to 2016.

**Methods:**

We collected data from a cohort of 395 houses in Pueblo Nuevo, Nicaragua. Primary data were collected through a field survey to assess post-intervention levels of *T*. *dimidiata* house infestation in 2016, two years after the large-scale insecticide spraying. We obtained secondary data from the records about past infestation levels and control activities between 2010 and 2015. Multilevel mixed-effects logistic regression analyses were used to identify factors associated with post-intervention house infestation.

**Results:**

The control program effectively reduced the infestation level from 2010 to 2014. Community-based surveillance was introduced in 2013; however, post-intervention infestation in 2016 had nearly reached pre-intervention levels in rural villages. Post-intervention house infestation was positively associated with poor wall construction, roofing tiles piled in the peri-domestic areas or the presence of dogs. Interestingly, the odds of post-intervention house infestation were one-fifth less when villagers sprayed their own houses regularly. Past infestation levels and the intensity of government-led insecticide spraying did not explain post-intervention house infestation.

**Conclusions:**

The vector control program failed to offer sustained reductions in *T*. *dimidiata* house infestation. This experience would suggest that community-based surveillance is an insufficient approach to suppressing *T*. *dimidiata* house infestation over many years. This study provides evidence to suggest that control policies for *T*. *dimidiata* should be reconsidered throughout Central America.

## Introduction

Chagas disease is caused by the parasite *Trypanosoma cruzi*. It is one of the most important neglected tropical diseases in Latin America in terms of the prevalence and healthy life years lost [1]. While the disease has been recognized as a global health challenge recently, it has affected the rural poor in Latin America for many years [2]. In 2010, it was estimated that 5–6 million people were infected and around 13% of the population at risk of infection in Latin America [3]. Cardiomyopathy is the most important chronic manifestation of the disease and develops in 20–30% of infected individuals [4,5]. *T*. *cruzi* is transmitted by the feces of the triatomine vector as well as transfusion, contaminated food or drink and congenital transmission [6,7]. Among several transmission routes, vector-born transmission has the largest impact in Latin America and is also responsible for maintaining the life cycle of the parasite [8].

In Central America, two vector species, *Rhodnius prolixus* and *Triatoma dimidiata*, were considered important targets for vector control [9]. In 1997, El Salvador, Guatemala, Honduras and Nicaragua established a common goal of eliminating *R*. *prolixus* and reducing domestic population of *T*. *dimidiata* [10,11]. Elimination of *R*. *prolixus* was considered feasible because they were found exclusively in human dwellings in Central America [9,12]. The countries adopted and implemented a three-phase vector control strategy including, (i) a preparatory phase for estimating and mapping vector distribution; (ii) an attack phase for conducting several rounds of large-scale insecticide spraying of houses and (iii) a surveillance phase for the detection and treatment of residual foci of vectors [13]. This strategy practically eliminated *R*. *prolixus* from Central America and reduced the incidence of Chagas disease to one-seventh in the region [14]. After the successful control of *R*. *prolixus*, *T*. *dimidiata* has become the main target of the vector control programs.

*T*. *dimidiata* has a diverse ecological niche including domestic and sylvatic habitats in Central America [15]. This ecological diversity complicates the control of domestic populations of *T*. *dimidiata*. Literature has shown that *T*. *dimidiata* house infestation is associated with poor house constructions such as unplastered walls and dirt floors [16–21]; hiding sites in peri-domestic areas such as rock piles [21]; presence of animals around the houses such as dogs, cats, and mice [19,21]; and environmental factors such as temperature, humidity, pattern of croplands and vegetation [16,22]. Socioeconomic status [18,20] and common practices such as wall plastering [20,23] or fumigation of dwellings [24] may also affect *T*. *dimidiata* house infestation. In addition to these multiple factors, the ability of *T*. *dimidiata* to colonize human dwellings repeatedly even after the initial insecticide spraying perplexes vector control programs [7].

Despite the anticipated difficulty, experts have generally agreed that elimination of *T*. *cruzi* transmission by *T*. *dimidiata* would be achievable by implementing a three-phase vector control strategy, because the community-based surveillance after the large-scale insecticide spraying would allow for re-treatment of newly infested houses [9,12,25]. Based on this expert view, El Salvador, Guatemala, Honduras and Nicaragua have implemented the three-phase vector control programs against *T*. *dimidiata* during the period of 2000–2015. Evaluations of the large-scale spraying programs have consistently shown that the spraying interventions reduced *T*. *dimidiata* house infestation in the short run, while persistence or reemergence of *T*. *dimidiata* house infestation was also observed after the insecticide spraying [26–29]. The four countries then introduced community-based vector surveillance to sustain the reduced level of *T*. *dimidiata* house infestation [30,31]. Yet, the effect of vector surveillance on *T*. *dimidiata* house infestation is not clearly understood. No study has examined the long-term trends of *T*. *dimidiata* house infestation throughout the three-phase vector control programs.

This paper aims to examine the long-term effectiveness of the three-phase vector control programs on *T*. *dimidiata* house infestation in Nicaragua. A long-term assessment is necessary since *T*. *dimidiata*’s capacity to infest human dwellings repeatedly can undermine initial gains of the vector control programs. In assessing empirical data from Nicaragua, we critically review the program assumption that community-based surveillance can suppress *T*. *dimidiata* house infestation for many years. This assumption has been a critical basis for the vector control programs in Central America for two decades. In addition, we will intend to explain *T*. *dimidiata* house infestation during the post-intervention period. Based on the findings, we discuss the limitations of the current vector control approach and suggest a future direction for control of *T*. *dimidiata* in Central America.

## Methods

### Site

Nicaragua is a Central American country that implemented the three-phase vector control program for Chagas disease. The country started vector control efforts during 1998–2000, but only in areas infested by *R*. *prolixus* [32]. Control of *T*. *dimidiata* has been widely pursued since the Nicaraguan Ministry of Health (MoH) and Japan International Cooperation Agency (JICA) began a bilateral technical cooperation project in 2009. The MoH/JICA project targeted *T*. *dimidiata* in five northern departments, namely Estelí, Jinotega, Madriz, Matagalpa and Nueva Segovia. The MoH/JICA cooperation ended in 2014 but MoH sustained control efforts even after it.

**Fig 1:**
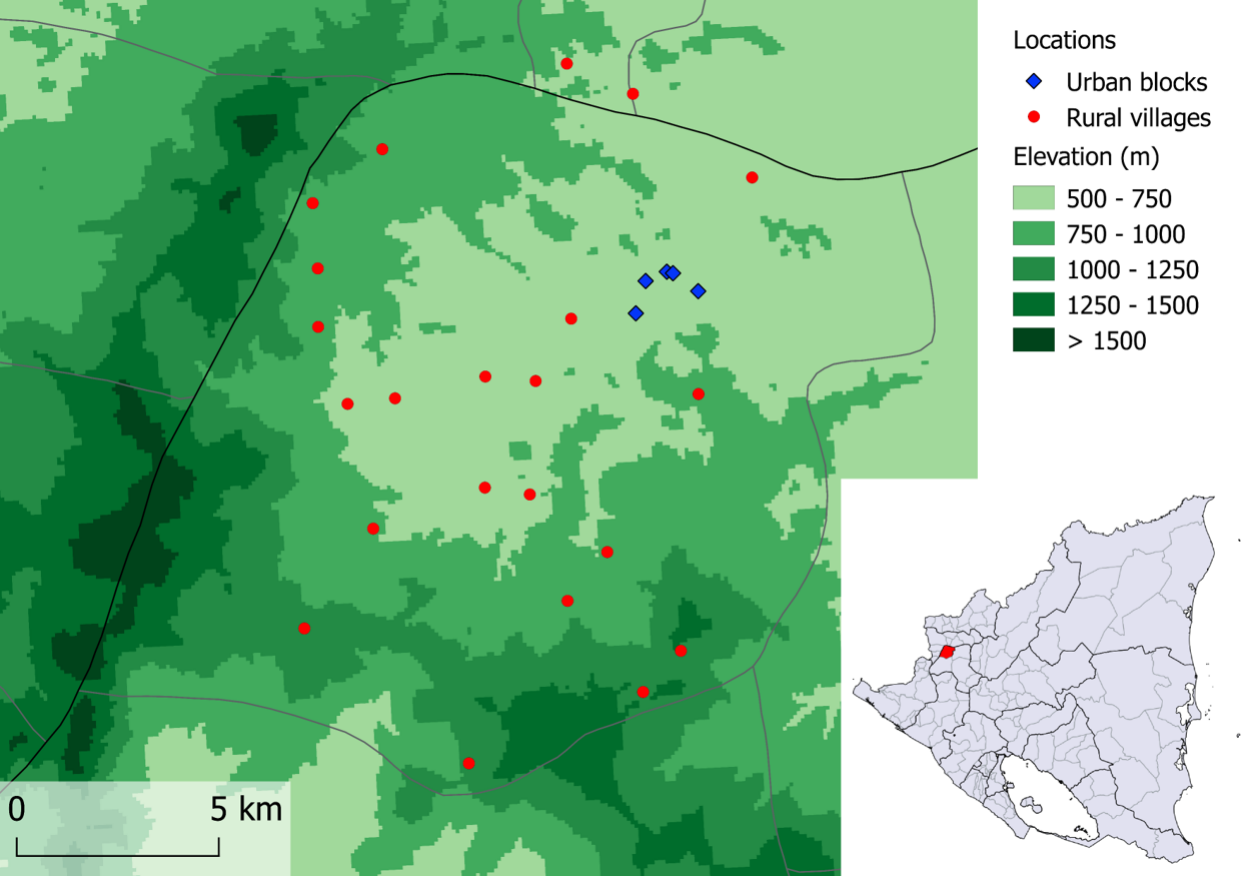
Locations of the municipality of Pueblo Nuevo (indicated in the small country map) and 27 villages/blocks involved in the present survey. Note: The authors created the figure using QGIS software ver 2.18.7. Administrative areas and elevation data are from the opensource DIVA-GIS (http://www.diva-gis.org/gdata). Point data are collected by the authors.

This study focuses on the Municipality of Pueblo Nuevo, Department of Estelí (Fig 1). In this municipality, there were no formal Chagas disease vector control activities until the MoH/JICA project began in 2009. The municipality experienced the full course of the three-phase vector control program: a baseline survey of *T*. *dimidiata* house infestation in 2010 (preparatory phase), three rounds of large-scale insecticide spraying in 2011 and 2014 (attack phase) and community-based vector surveillance since 2013 to the present (surveillance phase). In addition, the MoH/JICA project did an endline survey in 2014 to evaluate the level of *T*. *dimidiata* house infestation at the end of the project. Since the MoH/JICA project launched, the Municipal Health Center recorded every vector control activity. No other initiatives, organizations or programs worked for Chagas disease control in Pueblo Nuevo during the MoH/JICA project period. According to the Municipal Health Center, Pueblo Nuevo has approximately 23,500 people living in 5,800 houses that are geographically distributed among 49 rural villages and 10 urban blocks. Urban blocks are distinguished from rural villages as they are located within the municipal capital and characterized by planned streets, electricity and commercial establishments. The municipal capital is located at around 600 m above sea level.

### Primary data collection

**Fig 2:**
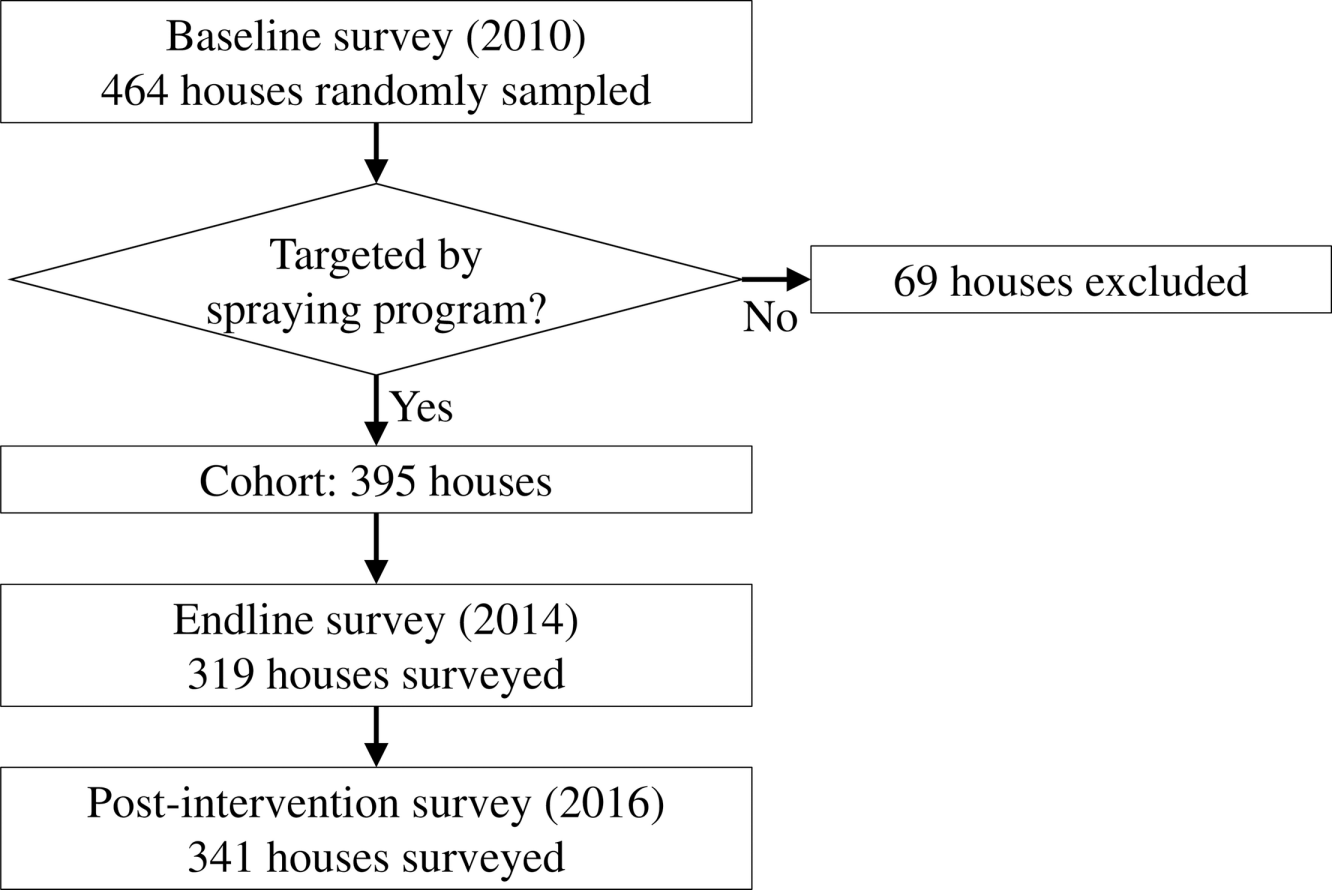
Selection of a 395-house cohort and flow of three surveys.

We conducted a field survey in 2016 to measure the post-intervention level of *T*. *dimidiata* house infestation and house-level covariates. This 2016 post-intervention survey targeted a cohort of 395 houses in 27 villages/blocks, which are a subset of random sample defined by the 2010 baseline survey (Fig 2). The baseline survey applied a two-step random sampling: (i) a probability proportional to size sampling to select 30 survey locations in the municipality and (ii) a segmentation sampling to select randomly one reference house and adjacent houses in each survey location. As a result, the baseline survey obtained data from 464 houses in the Municipality of Pueblo Nuevo. During the following attack phase, 69 houses were excluded from the large-scale spraying because they belonged to urban blocks where the risk of *T*. *dimidiata* house infestation was considered specially low [28]. Since our purpose is to assess the pattern of *T*. *dimidiata* house infestation in relation to the vector control program and not to obtain representative data to the entire municipality, we restricted our sample to the 395 houses. These houses were targeted by the baseline survey, the insecticide spraying, the 2014 endline survey and community-based vector surveillance, prior to the 2016 post-intervention survey.

For the field survey, we organized seven field teams, each consisting of two data collectors. Data collectors were trained for one day on Chagas disease, vector identification, data collection and reporting. The data collection was supervised directly by four supervisors, including two of the authors (KY and EP). Some of the data collectors and supervisors had experience of carrying out prior surveys. To minimize inter-observer bias, we shuffled team members every two or three days.

Houses were identified using the unique code assigned by the MoH/JICA project during the 2010 baseline survey. House identification was complemented by confirming householders’ names as registered during the previous two surveys. In each house, the data collectors requested informed consent from householders before obtaining any data. In the case where householders were absent, we rescheduled the house survey for up to two additional visits. When householders selected not to participate in the survey or the houses were uninhabited or relocated, these houses were skipped in the survey without replacement.

To detect *T*. *dimidiata* house infestation, we applied the standardized man-hour manual search method [33]. The previous two surveys used the same entomological technique; thus, the data from all three surveys are technically comparable. In each house, two data collectors spent 30 minutes for searching *T*. *dimidiata* using flashlights and tweezers in both of intra- and peri-domestic areas. Priority was given to the inside of bedrooms, where *T*. *dimidiata* is more likely to be found [34]. When *T*. *dimidiata* were found, the data collectors captured bugs in plastic bags. The supervisors double-checked species of the captured bugs to avoid taxonomic misidentification. The site of bug capture and bug’s developmental stage were recorded.

Following the entomological inspection, the data collectors recorded physical house conditions through direct observation, including predominant house construction materials, potential risk-factors in peri-domestic areas as well as possession of domestic animals. They also conducted a structured interview to obtain data from the householders about the presence of animals inside the house, their preventive behaviors and age of house construction. Furthermore, they collected socioeconomic data from householders. All data were recorded in paper formats, which were then digitalized by a data entry operator and cross-checked by the authors (KY and EP).

### Secondary data collection

We also collected secondary data from the MoH/JICA project archive to review the history of vector control program as well as to construct village-level covariates. We obtained digital databases of the 2010 baseline survey, the large-scale insecticide spraying in 2011 and 2014, the vector surveillance from 2012 to 2015 and the 2014 endline survey. These databases were summarized to outline all interventions undertaken in Pueblo Nuevo. We calculated three village-level explanatory variables: 1) house infestation index (= number of houses infested by *T*. *dimidiata* / number of houses inspected x 100) at the 2010 baseline survey, 2) the same index at the 2014 endline survey, and 3) number of spray rounds carried out during the large-scale insecticide spraying.

### Variables

Using primary and secondary data, we constructed one outcome variable, 19 explanatory variables and two control variables. The outcome variable is a binary indicating whether *T*. *dimidiata* was found in a house at the time of the 2016 post-intervention survey.

The explanatory variables consist of 16 house-level and three village-level variables. The 16 house-level explanatory variables were measured in the 2016 post-intervention survey, summarized in four categories:

- *house construction materials*; predominant wall material (categorical, 1- concrete or block, 2- plastered adobe, 3- adobe without plastering, 4- bajareque or wood), predominant roof material (categorical, 1- zinc, 2- tiles, 3- others) and predominant floor material (categorical, 1- cement or tiles, 2- dirt, 3- others),
- *peri-domestic conditions*; presence of rock fences (binary), presence of piled roofing tiles (binary) and presence of piled firewood (binary),
- *domestic animals*; presence of dogs (binary), presence of cats (binary), presence of pigs (binary), presence of chickens in henhouse (binary), presence of chickens without henhouse (binary), reported presence of any animals sleeping inside the house (binary) and reported presence of rats inside the house (binary),
- *residents’ preventive behaviors*; whether the walls are plastered more than once every 30 days (binary) and whether the walls are sprayed more than once every 30 days (binary).

The village-level explanatory variables represent the history of vector infestation and control. These involve the *T*. *dimidiata* house infestation indices at the 2010 baseline survey (continuous), the house infestation indices at the 2014 endline survey (continuous) and the number of spray rounds implemented by the MoH/JICA project during 2011–2014 (count).

We used two house-level control variables. The first one is the categorical age of the house (1- less than or equal to 10 years, 2- between 10 and 20 years, 3- more than 20 years). The second one is the poverty score that we calculated based on the seven socioeconomic conditions. We assigned one point for every following condition: if there were more than three persons per bedroom, if there were more than three dependents per person with an income, if the householder did not own a plot for sowing, if the householder did not own any electronic equipment, if there was no drinking water available in the house, if there was no latrine in the house, and if no shower was available for bathing. The sum of the points was averaged by the number of conditions answered and then multiplied by 10 so that the poverty score could range between 0 (least poor) and 10 (poorest).

### Data analysis

We first illustrated the longitudinal trends in *T*. *dimidiata* house infestation among three surveys using line graphs. Based on the data from the 395-house cohort, the house infestation indices were calculated separately for urban and rural areas at three points in time. Adopting the definition of the Nicaraguan government, urban blocks refer to residential areas in the municipal capital, characterized by planned streets, electric services and commercial establishments. The 2014 endline survey and 2016 survey could not inspect all houses in the cohort. In the data analysis, we assumed missingness completely at random. For each data point, 95% Wilson confidence intervals were calculated. In addition, we created a 2x2 table to analyze the composition of *T*. *dimidiata* captured in the 2016 survey, by the site of bug capture and bug’s developmental stage.

Next, we analyzed factors associated with *T*. *dimidiata* house infestation observed in the 2016 post-intervention survey. We restricted our regression analyses to 280 houses in 22 rural villages given that no house infestation was observed in urban blocks during the 2016 survey. Since *T*. *dimidiata* can migrate between houses [35,36], houses within the same village are not independent. To deal with the intra-cluster correlation, we selected a multilevel mixed-effects model with houses as the primary level and villages as the secondary level of analysis. We assumed a random variance in intercepts between villages. For each explanatory variable, a univariable multilevel mixed-effects logistic regression analysis was employed to calculate an unadjusted odds ratio (OR).

Finally, we constructed two models using multivariable multilevel mixed-effects logistic regression to calculate adjusted odds ratios (aOR). We reduced the number of explanatory variables included in the regression models by selecting the house-level explanatory variables that had statistically significant associations in the univariable analyses. In the first model, we assessed only the selected house-level explanatory variables collected in the 2016 survey. Using this model, we assessed whether two control variables (i.e. categorical age of the house and poverty score) confound the effects of explanatory variables on *T*. *dimidiata* house infestation. We also assessed multicollinearity by using uncentered variance inflation factors. In the second model, we added the village-level explanatory variables to assess the effect of prior vector infestation and control. A likelihood-ratio test was used to assess whether the village-level variables better explain *T*. *dimidiata* house infestation. We also evaluated the goodness-of-fit of the two models using concordance statistics by calculating an area under the receiver operating characteristic curve (AUC). All statistical analyses were conducted at a significance level of 5% using Stata Statistical Software Release 14.2. (StataCorp LP, College Station, TX).

### Ethical considerations

The ethical aspects of this study were reviewed and approved by the ethical committee of Nagasaki University, Japan (Ref. 75, May 29, 2015). The Ministry of Health in Nicaragua also authorized this study (DGD-JMCM-1065-08-15, August 17, 2015). Our study combines routine data collected by the JICA/MoH project and original data collected by our field survey in 2016. During the field survey, the householders who agreed to participate in the study provided written informed consent.

## Results

### Review of vector control program

The secondary data allowed us to describe all activities related to the Chagas disease vector control program in Pueblo Nuevo. The 2010 baseline survey assessed a random sample of 464 houses, among which 82 (17.7%) were found to be infested by *T*. *dimidiata*. Given the relatively high level of *T*. *dimidiata* house infestation, this municipality was included in the attack phase. The attack phase involved three rounds of large-scale spraying, treating 3,699 houses in 46 villages/blocks (1^st^ round between January and August 2011), 3,741 houses in 42 villages/blocks (2^nd^ round between January and April 2013) and 1,791 houses in 24 villages/blocks (3^rd^ round between November and December 2013). The spraying program used one of two types of pyrethroid-derivative insecticide (etofenprox 20% wettable powder or alpha-cypermethrin 10% wettable powder). The large-scale spraying significantly decreased the level of *T*. *dimidiata* house infestation in the program area [28]. The endline survey between March and Abril in 2014 assessed 319 houses, among which 19 (6.0%) were found to be infested by *T*. *dimidiata*.

The surveillance phase was introduced in January 2013 to sustain the low level of *T*. *dimidiata* house infestation. Following MoH guidelines [37], the Municipality of Pueblo Nuevo established a community-based vector surveillance system. In this surveillance system, community residents capture and report bugs to the nearest health facilities, and then health staff provides responses such as educational house visits or insecticide spraying (for details, see Yoshioka et al. [31]). From 2013 to 2015, the municipal health authority received *T*. *dimidiata* reports from 46–89 houses per six-month period (Table 1). The responses by the health staff were operationally satisfactory. Expected response rates were over 80% for house visits and 50% for insecticide spraying, per the criteria of the MoH/JICA project. Table 1 shows the response rates met these requirements in most of the six-month periods. The vector surveillance in Pueblo Nuevo worked as expected by the MoH/JICA project.

**Table 1:** Results of the vector surveillance-response system in Pueblo Nuevo, Estelí, Nicaragua.

Type of response	2013		2014		2015	
	Jan-Jun	Jul-Dec	Jan-Jun	Jul-Dec	Jan-Jun	Jul-Dec
Educational house visit						
Houses with bug reports	71	52	89	46	83	59
Houses visited	58	32	89	45	70	48
Response rate (%)	81.7%	61.5%	100.0%	97.8%	84.3%	81.4%
Insecticide spraying						
Houses eligible for spraying*	2	8	14	5	4	20
Houses sprayed	0	2	10	5	0	16
Response rate (%)	0.0%	25.0%	71.4%	100.0%	0.0%	80.0%

* Eligibility criteria are defined by the national guideline (see Yoshioka et al. [31]).

### Trends of *T*. *dimidiata* house infestation

The post-intervention field survey was carried out in February 2016, almost two years after the 2014 endline survey. The 2016 survey targeted a cohort of 395 houses in 27 villages/blocks, of which 341 (86.6%) were examined by the field data collection teams. We could not inspect 54 houses, of which 31 were uninhabited, 12 were relocated to other places, eight were closed (i.e. householders were absent at the time of house visits), two rejected participation in the survey and one could not be identified. Among 341 houses assessed, 41 (12.0%) were infested by *T*. *dimidiata*.

**Fig 3:**
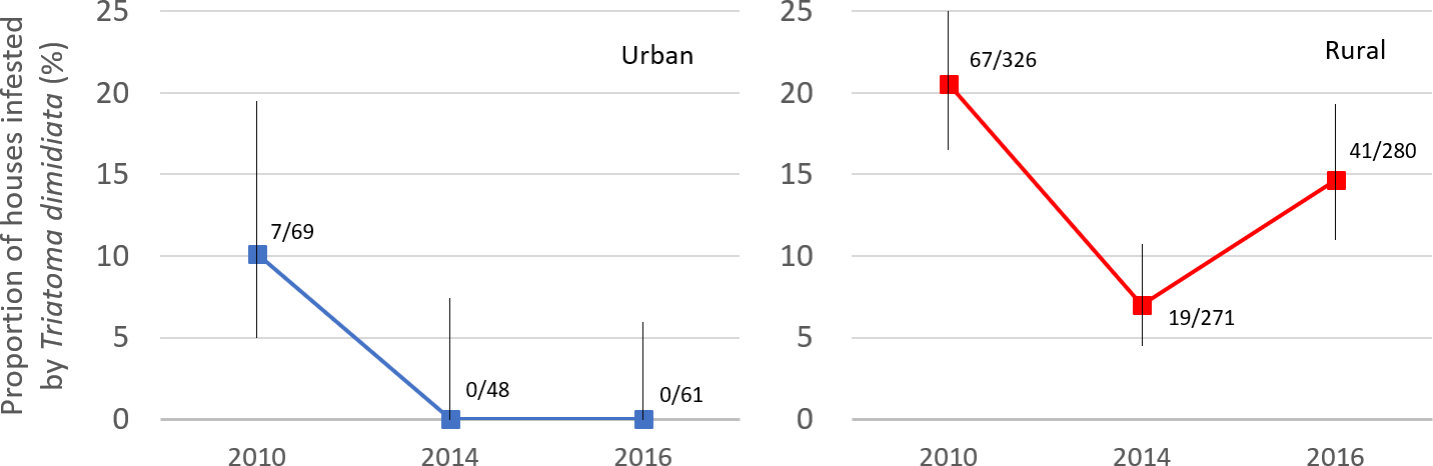
Trends of *T*. *dimidiata* house infestation in urban and rural areas, Pueblo Nuevo, Nicaragua. The graph areas show the number of houses infested / houses investigated. The vertical bars represent 95% Wilson confidence intervals.

Fig 3 shows the trends of *T*. *dimidiata* house infestation among the cohort of 395 houses in Pueblo Nuevo, separately for urban and rural areas. Data were obtained from the 2010 baseline survey (n = 395), the 2014 endline survey (n = 319) and the 2016 post-intervention survey (n = 341). In 2010, the house infestation indices were as high as 10.1% and 20.6% in urban and rural areas, respectively. After the large-scale insecticide spraying during 2011–2013, the 2014 endline survey found that the house infestation indices decreased drastically to 0.0% and 7.0%, respectively. The 2016 survey showed the *T*. *dimidiata* house infestation did not recover in urban areas; however, the index increased by 7.6%-points in rural areas in two years.

Table 2 shows the composition of *T*. *dimidiata* captured in the 2016 survey, classified by site of bug capture and bug’s developmental stage. Among 95 specimens captured, more than half of them were found in peri-domestic areas, although we prioritized the bug search in bedrooms. In addition, we found twice as many nymphs as adults, even though the man-hour manual search tends to find more adults because of their visibility. In total, about 40% of the captured bugs were nymphs found in peri-domestic areas. Several peri-domestic colonies were identified during the survey, including a focus where four adults and 17 nymphs were found together.

**Table 2:** Numbers of *T*. *dimidiata* captured during the 2016 survey, by the site of bug capture and developmental stage.

		Developmental stage		Total
		Adult	Nymph	
Site of bug capture	Intra-domestic	19 (20.0%)	22 (23.2%)	41 (43.2%)
	Peri-domestic	13 (13.7%)	41 (43.2%)	54 (56.8%)
Total		32 (33.7%)	63 (66.3%)	95 (100%)

### Factors associated with vector house infestation

Fig 3 shows that the pattern of *T*. *dimidiata* house infestation was remarkably different between urban and rural areas in Pueblo Nuevo. To analyze the factors associated with *T*. *dimidiata* house infestation in the post-intervention period, we restricted our statistical analyses to rural areas where *T*. *dimidiata* reinfestation was observed in the 2016 survey.

Table 3 presents descriptive statistics of all variables and univariable analyses of 21 covariates. The 2016 survey found 41 houses (14.6%) infested by *T*. *dimidiata* in rural areas. While nearly half of the houses are constructed with concrete or blocks as wall materials, 10.7% of the houses have poorest wall materials such as bajareque (wood sticks interwoven with reeds and mud). The poorest wall materials are associated with a significantly increased odds of vector infestation compared to the concrete or block walls. In the peri-domestic areas, the presence of piled roofing tiles shows significant association with *T*. *dimidiata* house infestation. Among domestic animals, the presence of dogs is significantly associated with *T*. *dimidiata* house infestation. Houses are strongly protected from *T*. *dimidiata* infestation when the residents apply insecticide to their houses more than once every 30 days. Odds of *T*. *dimidiata* infestation increase significantly when the houses are more than 10 years old.

**Table 3:** Descriptive statistics and univariable multilevel mixed-effects logistic analyses for 280 houses located in rural villages.

Variables		Category / Range	Freq.	Percentage / Mean	Unadjusted OR (95%CI)	
***Outcome variable***						
	1. House infested by *T*. *dimidiata*	No	239	85.4%	-	
		Yes	41	14.6%	-	
***House construction***						
	2. Predominant wall material	Concrete or block	131	46.8%	Ref.	
		Adobe (plastered)	93	33.2%	1.09 (0.46, 2.58)	
		Adobe (not plastered)	26	9.3%	2.11 (0.64, 6.94)	
		Bajareque or wood	30	10.7%	3.63 (1.17, 11.2)	*
	3. Predominant roof material	Zinc	175	62.7%	Ref.	
		Tiles	102	36.6%	1.68 (0.80, 3.54)	
		Others	2	0.7%	Omitted	
	4. Predominant floor material	Cement or tiles	94	33.8%	Ref.	
		Dirt	176	63.3%	1.79 (0.80, 3.98)	
		Others	8	2.9%	1.09 (0.11, 10.5)	
***Peridomestic conditions***						
	5. Presence of rock fences	No	245	87.5%	Ref.	
		Yes	35	12.5%	1.83 (0.68, 4.89)	
	6. Presence of piled roofing tiles	No	197	70.4%	Ref.	
		Yes	83	29.6%	2.80 (1.34, 5.85)	*
	7. Presence of piled firewood	No	154	55.0%	Ref.	
		Yes	126	45.0%	1.36 (0.67, 2.78)	
***Domestic animals***						
	8. Presence of dogs	No	76	27.1%	Ref.	
		Yes	204	72.9%	6.26 (1.80, 21.7)	*
	9. Presence of cats	No	185	66.1%	Ref.	
		Yes	95	33.9%	1.90 (0.94, 3.84)	
	10. Presence of pigs	No	189	67.5%	Ref.	
		Yes	91	32.5%	1.88 (0.92, 3.84)	
	11. Presence of chickens in henhouse	No	220	78.6%	Ref.	
		Yes	60	21.4%	0.70 (0.28, 1.72)	
	12. Presence of chickens without henhouse	No	119	42.5%	Ref.	
		Yes	161	57.5%	1.72 (0.83, 3.56)	
	13. Any animals sleeping inside the house	No	233	84.1%	Ref.	
		Yes	44	15.9%	1.30 (0.53, 3.18)	
	14. Reported presence of rats inside the house	No	94	33.7%	Ref.	
		Yes	185	66.3%	1.24 (0.59, 2.62)	
***Residents' behavior***						
	15. Plaster walls more than once every 30 days	No	257	92.5%	Ref.	
		Yes	21	7.6%	0.55 (0.17, 2.56)	
	16. Spray more than once every 30 days	No	199	71.1%	Ref.	
		Yes	81	28.9%	0.21 (0.07, 0.63)	*
***Control variables***						
	18. Age of house construction in year	≤ 10	76	27.1%	Ref.	
		> 10, ≤ 20	109	38.9%	4.04 (1.32, 12.4)	*
		> 20	95	33.9%	3.96 (1.26, 12.5)	*
	19. Poverty score	0 to 8.6	280	3.1	0.88 (0.72, 1.07)	
***Village-level variables***^**†**^						
	20. House infestation index at baseline (%)	4.3 to 71.4	22	21.3	1.01 (0.99, 1.04)	
	21. House infestation index at endline (%)	0.0 to 25.0	22	7.6	0.98 (0.92, 1.05)	
	22. Number of spray rounds	1 to 3	22	2.3	1.12 (0.56, 2.21)	

Odds ratios are calculated by mixed-effects logistic regression using villages as a group variable

† Means are averages of the village-level values. Odds ratios are computed using houses as a unit of analysis.

* p-value < 0.05

Adjusted odds ratios (aOR) are calculated by two models using the multivariable multilevel mixed-effects logistic regression (Table 4). Model 1 included four house-level explanatory variables whose unadjusted ORs were significant in the univariable analyses in addition to two control variables. In assessing the confounders, we found evidence that both age category of house construction and poverty score confounds the effects of wall materials on *T*. *dimidiata* house infestation. Thus, these two control variables were kept in our regression models. No evidence of multicollinearity was found. The goodness-of-fit analysis shows that Model 1 has excellent discrimination (AUC = 0.899). The random intercept parameter is statistically significant and likelihood-ratio test comparing Model 1 to the model without multilevel specification is also significant (χ^2^_(1)_ = 9.06, p-value = 0.001). The last two statistics support the assumption that houses are not independent within a village, justifying the use of multilevel mixed-effects modelling.

**Table 4:** Multivariable, multilevel mixed-effects logistic regression analyses examining the factors associated with *T*. *dimidiata* house infestation among 280 houses located in 22 rural villages.

Covariates			Model 1		Model 2	
			aOR (95%CI)		aOR (95%CI)	
**House-level covariates**						
	Predominant wall material					
		Concrete or block	Ref.		Ref.	
		Adobe (plastered)	1.13 (0.41, 3.15)		1.14 (0.41, 3.20)	
		Adobe (not plastered)	2.85 (0.67, 12.1)		2.91 (0.68, 12.5)	
		Others (bajareque or wood)	4.46 (1.20, 16.6)	*	4.41 (1.18, 16.5)	*
	Presence of piled roofing tiles		3.54 (1.45, 8.63)	*	3.70 (1.51, 9.06)	*
	Presence of dogs		8.89 (2.30, 34.4)	*	9.10 (2.33, 35.6)	*
	Spray more than once every 30 days		0.15 (0.04, 0.51)	*	0.16 (0.05, 0.54)	*
	Age of house construction in year					
		≤ 10	Ref.		Ref.	
		> 10, ≤ 20	5.85 (1.59, 21.5)	*	5.83 (1.59, 21.4)	*
		> 20	3.62 (0.98, 13.4)		3.69 (1.01, 13.5)	*
	Poverty score		0.87 (0.68, 1.11)		0.87 (0.68, 1.12)	
**Village-level covariates**						
	House infestation index at baseline (%)		-		1.01 (0.97, 1.05)	
	House infestation index at endline (%)		-		0.95 (0.86, 1.05)	
	Number of spray rounds		-		0.88 (0.28, 2.74)	
**Random intercept parameter**						
	Variance between villages		1.27 (0.37, 4.40)	*	1.20 (0.34, 4.26)	*
ICC			0.279		0.268	
AUC			0.899		0.898	
Likelihood-ratio test						
	vs. single-level model (d.f. = 1)		8.73	*	9.06	*
	vs. Model 1 (d.f. = 3)		-		1.03	

* p < 0.05.

ICC: Intracluster correlation coefficient.

AUC: Area under the ROC curve.

Model 1 shows that the poor wall materials (bajareque) significantly increase the odds of *T*. *dimidiata* house infestation, compared to the houses with concrete or block walls (aOR: 4.46; 95%CI: 1.20–16.6), after adjusting for other covariates included in the model. The presence of piled roofing tiles in the peri-domestic areas is also positively associated with *T*. *dimidiata* house infestation (aOR: 3.54; 95%CI: 1.45–8.63). The presence of dogs increases the odds of house infestation by almost nine times (aOR: 8.89; 95%CI: 2.30–34.4). Residents’ insecticide spraying behavior is a strong protective factor, decreasing the odds of *T*. *dimidiata* house infestation to one-fifth (aOR: 0.21; 95%CI: 0.06–0.67).

Model 2 adds three village-level explanatory variables to Model 1 to examine the role of the past house infestation levels and the intensity of spraying program. Model 2 shows that the past house infestation levels and the number of spray rounds applied to each village are not significantly associated with the post-intervention *T*. *dimidiata* house infestation. Compared to Model 1, there are only marginal changes in aORs for house-level covariates. The goodness-of-fit analysis shows no improvement in the model’s discriminatory capability (AUC = 0.898). Likelihood-ratio test, comparing Model 2 to Model 1, is not statistically significant (χ^2^_(3)_ = 1.03, p-value = 0.795). These statistics all agree that the past infestation levels and the intensity of spraying program are not important to explain the post-intervention *T*. *dimidiata* house infestation. The random intercept term remains statistically significant in Model 2, implying there could be some unobserved village-level variables which can explain *T*. *dimidiata* house infestation.

## Discussion

This is the first study to examine the long-term patterns of *T*. *dimidiata* house infestation in relation to the vector control programs in Central America. We found that the vector control program implemented in Pueblo Nuevo has failed to control *T*. *dimidiata* house reinfestation sustainably. Shortly after the attack phase, *T*. *dimidiata* house infestation successfully dropped below 7% both in urban and rural settings. During the subsequent surveillance phase, low house infestation levels were sustained in urban areas; however, *T*. *dimidiata* house infestation quickly recovered towards the pre-intervention levels in rural areas even though the vector surveillance performed acceptably. These two discrete scenarios of *T*. *dimidiata* house infestation in urban as compared to rural areas must be noted in understanding the long-lasting effects of the vector control program, with special attention to the rural areas.

**Fig 4:**
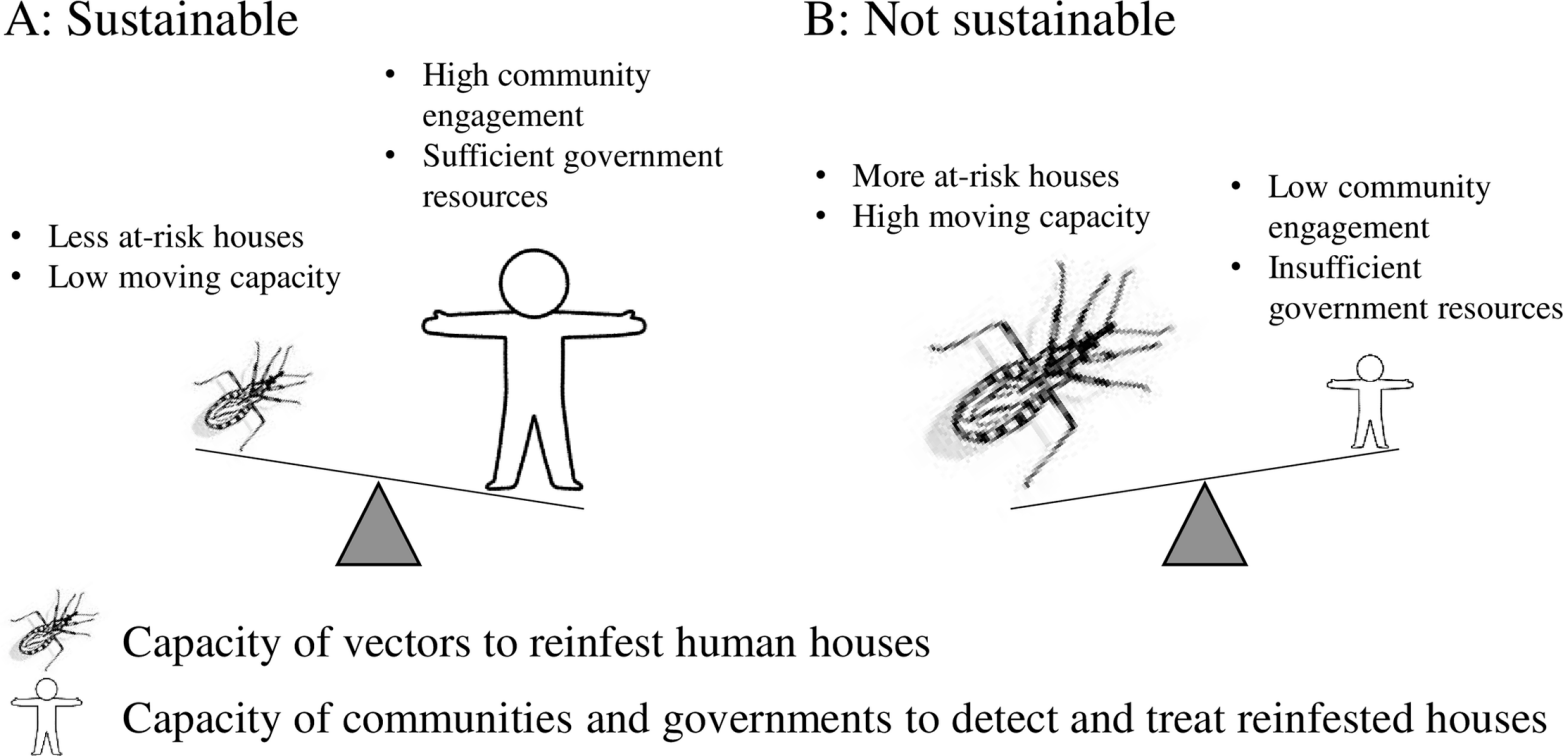
Two conceptual scenarios where the interruption of vector-borne *T*. *cruzi* transmission could be sustained (A) and could not be sustained (B) through community-based vector surveillance.

Vector control experts have claimed that the three-phase vector control program is an effective means of controlling *T*. *dimidiata*, under the assumption that the community-based surveillance enables selective control wherever the vectors attempt to recolonize houses [9,12,25]. Our evidence shows that this assumption did not become a reality in Pueblo Nuevo. The failure is largely attributable to the practical limits of the community-based surveillance. A previous pilot study in Nicaragua suggested that the community-based surveillance would inevitably involve unreported vectors, delayed responses and limited government capacity of insecticide spraying [31]. Given the vast distribution of *T*. *dimidiata* and the under-resourced health system, the community-based surveillance has only a partial capacity to detect and treat *T*. *dimidiata* house infestation. Based on these arguments, we propose to conceptualize that the interruption of vector-borne *T*. *cruzi* transmission as something which cannot be sustained when the resilience of vectors exceeds the capacity of the community-based surveillance (Fig 4). Our concepts would be applicable not only to Pueblo Nuevo but also to many rural villages in Guatemala, Honduras, El Salvador and Nicaragua where the *T*. *dimidiata* is similarly resilient to reinfest human houses and the communities and governments have limited resources.

We demonstrated that the community-based surveillance cannot maintain *T*. *dimidiata* house infestation at a reduced level. However, this does not mean that the surveillance must be discontinued but rather that its role should be reconsidered as part of more holistic approach. The benefits of community-based surveillance include that it can collect entomological data at a low cost on a regular basis in order to identify high-risk areas [31]. In addition, the house visits conducted by health service providers to respond vector reports would contribute to creating a solid knowledge base about Chagas disease among rural populations. Since Chagas disease is not a well-understood problem, improvement of local knowledge is crucial to promote prevention. Therefore, we propose to continue the community-based surveillance of *T*. *dimidiata* and to strengthen or complement the current approach by incorporating the following recommendations.

For improving control efforts against *T*. *dimidiata*, it is essential to understand the mechanisms of *T*. *dimidiata* reinfestation. Our study suggests peri-domestic conditions serve as important sources of *T*. *dimidiata* reinfestation. In our study, 43% of bugs captured were nymphs in peri-domestic areas (Table 2). This figure would be unusually high, because *T*. *dimidiata* are usually concentrated in bedrooms [34] and the manual search method used in the study tends to find fewer nymphs simply due to their smaller size and lower visibility. This unusually high rate implies that *T*. *dimidiata* is likely to colonize first the peri-domestic areas in the process of recovering their domestic population after the large-scale insecticide spraying. Our multivariable regression analyses also support this hypothesis, by showing that the presence of piled roofing tiles in peri-domestic areas is associated with three times the odds of finding *T*. *dimidiata* infestation (Table 4). It is known that the objects in the peri-domestic conditions serve as artificial ecotopes for *T*. *dimidiata* [38]. In general, insecticide loses its residual effects quickly in peri-domestic areas, due to sunlight, high temperatures, rain and dust [39,40]. The importance of peri-domestic conditions as a source of reinfestation is suggested in case of *T*. *infestans* in South America [39,41,42] as well as *T*. *pallidipennis* and *T*. *barberi* in Mexico [43]. Serological screening of dogs may be useful as sentinels to detect reemerging vector-borne *T*. *cruzi* transmission in peri-domestic areas [44]. The control efforts must emphasize the importance of peri-domestic areas as sources of reinfestation and as focal points of surveillance.

Another way to complement the control efforts against *T*. *dimidiata* is to enhance community-based interventions. Evidence from our study shows regular insecticide spraying by residents is highly effective to prevent *T*. *dimidiata* house infestation. In our study site, some residents spray their own houses to control nuisance insects such as fleas or scorpions. We observed that they commonly applied cypermethrin formulated in emulsifiable concentrates. This type of insecticide is accessible in rural villages for agricultural use. Following to the agricultural standards, the residents often dilute 1–5 cc of cypermethrin with one liter of water, getting the maximum dosage of active ingredients at 0.05 g/m^2^. According to the WHO standards for vector control, the effective control of triatomines requires cypermethrin at 0.125 g/m^2^ [45]. Thus, the common dosage used by villagers seems not to reach the lethal dose for triatomines, but the frequent spraying of insecticide at sublethal levels could work as a repellent to prevent *T*. *dimidiata* house infestation. If so, the self-spraying practices could be used more widely as a solution to sustainable triatomine prevention. In northwest Argentina, intermittent use of insecticides by householders, coupled with local environmental management, is suggested as a sustainable approach [46]. Triatomines can develop insecticide resistance [47,48], but in theory, the sublethal use of insecticide causes no selection and would not contribute to the development of resistance. Yet, an unregulated long-term application must be carefully considered because it may promote overuse and further complicate the control of *T*. *dimidiata*.

Given that there are no effective and feasible instruments currently available to sustain interruption of *T*. *cruzi* transmission by *T*. *dimidiata*, the policy for Chagas disease control should be reformulated with an explicit recognition of active vector-borne *T*. *cruzi* transmission. Since 1997, PAHO/WHO’s policy and country-level actions have emphasized the elimination of vector-borne *T*. *cruzi* transmission [10]. This emphasis on prevention has inevitably overlooked the needs for treatment. Recognizing that the interruption of *T*. *cruzi* transmission by *T*. *dimidiata* is not sustainable, a future policy must enhance early diagnosis and treatment of Chagas disease patients.

Our study has several limitations. First, our study used non-random sample. The houses were located in relatively high-risk areas identified previously by the spraying program. Thus, the estimates of *T*. *dimidiata* house infestation presented in Fig 3 should not be interpreted as municipal representatives. Nonetheless, the temporal changes of *T*. *dimidiata* house infestation and their underlying causal patterns found in our sample provide important implications for the Chagas disease vector control programs in general. Second, interview data collected from householders are subject to recall bias. Some questions like frequency of self-spraying are likely to be biased, probably introducing measurement errors in our analysis. Third, our study is not designed to determine causal effects of the three-phase vector control program on *T*. *dimidiata* house infestation because the study does not involve a counterfactual analysis. For example, the study offers no answer to the question if the community-based surveillance is effective in slowing down the recovery of *T*. *dimidiata* house infestation. Despite this limitation, our study provides strong evidence that calls into question the assumption that has formed the foundation for vector control programs over the past several decades.

In summary, our study shows that *T*. *dimidiata* house infestation recovered quickly in rural Nicaraguan communities where the traditional three-phase vector control program has been successfully implemented. It was not realistic to expect that the community-based surveillance could detect and treat all *T*. *dimidiata* house infestation. Given the current entomological and social conditions, we strongly disagree with the idea that *T*. *cruzi* transmission by *T*. *dimidiata* can be eliminated in Central America using the current approach, despite the expectation on the part of international organizations that this would be possible. Interestingly, this study suggests that community efforts like regular self-spraying can be more effective than a government vector control program in preventing *T*. *dimidiata* house infestation. The control policy should be studied and revised on the premise that active transmission will continuously occur in Central America where *T*. *dimidiata* persists. A new policy should seek to balance community-based collective actions and institutional control programs and address both prevention and treatment.

## Supporting information

S1 File Survey questionnaire (in Spanish). (DOCX)

S2 File Survey questionnaire (translated in English). (DOCX)
