# Supplementary material for: The resilience of Triatoma dimidiata: An analysis of reinfestation in the Nicaraguan Chagas disease vector control program (2010–2016)
Source: PLoS One. 2018 Aug 23;13(8):e0202949. doi: 10.1371/journal.pone.0202949 (PMC6107243; doi:10.1371/journal.pone.0202949)
Supplement: S1 File — (DOCX) [file pone.0202949.s001.docx]

**HOJA DE CONSENTIMIENTO INFORMADO**

Estimado habitante de esta comunidad, mi nombre es: ________________________________________

Estamos realizando una encuesta para investigar la presencia de chinches, transmisores de la enfermedad de Chagas. La encuesta será conducida por la Universidad de Nagasaki en Japón, en colaboración con el Ministerio de Salud de Nicaragua -MINSA.

La información que Usted nos dé es **confidencial,** no será divulgada a ninguna persona que no tenga relación con esta encuesta y será utilizada solamente para analizar el riesgo de chinches en su comunidad,

Su participación es voluntaria y usted puede retirarse de la entrevista en cualquier momento o puede rehusar contestar cualquier pregunta.

Si está de acuerdo le pido me regale su firma de consentimiento indicando que usted está de acuerdo en participar en esta encuesta.

La forma de consentimiento ha sido leída y explicada al participante.

Firma de participante: _________________________________________________

Firma de encuestador: _________________________________________________

Fecha: _________/___Feb__/ 2016

| Información Básica  1-1. Fecha de la encuesta: _/ Feb / 2016  1-2. Código de la Casa:  1-3. Nombre de Comunidad/Barrio:  1-4. Nombre de Jefe de Familia:  1-5. ¿El nombre del jefe corresponde al que aparece en el listado? ☐ Sí ☐ No  1-6. ¿Hace cuentos años vive la familia en esa casa? ☐ Más de 6 años ☐ Menos de 5 años |
| --- |
| Coordenada (GPS) – UTM zona 16   \|  \|  \|  \|  \|  \|  \|  \| \| --- \| --- \| --- \| --- \| --- \| --- \| --- \| \|  \|  \|  \|  \|  \|  \|  \| |
| Material predominante del cuarto (observación del encuestador)  2-1. Pared: ☐ Bajareque sin repello ☐ Bajareque repellado ☐ Adobe sin repello  ☐ Adobe repellado ☐ Bloque/Ladrillo ☐ Concreto ☐ Madera  ☐Casa prefabricada loseta 　　☐ Otros:  2-2. Techo: ☐ Zinc ☐ Tejas ☐ Paja/Palma ☐ Otros:  2-3. Piso: ☐ Tierra ☐ Baldosado/Concreto ☐ Otros: |
| Condición de peri-domicilio (observación del encuestador)  3-1. Marque todo lo que se observa en el peri-domicilio (patio)  ☐ Cerco de piedra ☐ Acumulo de tejas ☐ Acumulo de leñas  ☐ Gallineros ☐ Chiqueros ☐ Perreras ☐ Jaula de pájaros  ☐ Otras cosas donde se pueden esconder los chinches: |
| Resultado de la Encuesta Entomológica  4-1. Tiempo (solo para buscar chinches, sin incluir el tiempo de llenar este cuestionario)  Hora que comienza :  Hora que termina :  4-2. Especie de chinches capturados:  ☐ *T. dimidiata* ☐ *R. prolixus* ☐ Otros:  4-3. Número de chinches y otras evidencias capturadas:   \| Lugar \| Capturados vivos \| \| Otras evidencias \| \| \| \| --- \| --- \| --- \| --- \| --- \| --- \| \| Adultos \| Ninfas \| Muertos \| Exuvia \| Feces \| \| Intra-domicilio \|  \|  \|  \|  \|  \| \| Peri-domicilio \|  \|  \|  \|  \|  \| \| Total \|  \|  \|  \|  \|  \| |

Observaciones:

Hoja de cuestionario al jefe (o su equivalente) de la vivienda

| 5-1. ¿Cuántos de cada animal tiene usted en su casa?  [anotar número] | Perros Gatos  Chanchos Pájaros  Gallinas en gallinero  Gallinas sin gallinero |
| --- | --- |
| 5-2. ¿Hay algún animal que duerme dentro de su casa? | ☐ Sí ☐ No ☐ No contesta |
| 5-3. ¿Ha visto ratas o ratones dentro de la casa? | ☐ Sí ☐ No ☐ No contesta |
|  |  |
| 6-1. ¿Usted o su familia repella la pared?  ¿Cada cuánto la repella? | ☐ Sí ☐ No ☐ No contesta  cada _______ días / semanas / meses |
| 6-2. ¿Usted o su familia fumiga la casa?  ¿Cada cuánto la fumiga? | ☐ Sí ☐ No ☐ No contesta  cada _______ días / semanas / meses |
|  |  |
| 7-1. (mostrar las fotos) ¿Usted conoce chinches? | ☐ Sí ☐ No ☐ No contesta |
| 7-2. ¿Ha visto chinches en su casa durante el año pasado? | ☐ Sí ☐ No ☐ No contesta |
| 7-3. ¿Ha entregado chinches al Centro de Salud durante el año pasado? | ☐Sí ☐ No ☐ No contesta  *Saltar a la pregunta “8-1”* |
| 7-4 ¿Cuántas veces ha entregado chinches el año pasado? | _______ veces |
| 7-5. ¿El personal o brigadista de salud ha visitado su casa después que usted entregó el chinche? | ☐ Sí ☐ No ☐ No contesta  *Saltar a la pregunta “8-1”* |
| 7-6. ¿Que personal lo visitó? | (Respuesta múltiples)  ☐ Médico ☐ Enfermero(a)  ☐ ETV ☐ Brigadista  ☐ Otro: _______________________________ |
| 7-7. Si el personal o brigadista de salud lo ha visitado después que usted entregó el chinche, ¿qué hizo en su casa? | (Respuesta múltiples)  ☐ Dio una charla sobre chinches  ☐ Revisó donde se encontraron los chinches  ☐ Fumigó la casa  ☐ Otro: _______________________________ |
|  |  |
| 8-1. ¿Hace cuantos años construyeron la casa? | años |
| 8-2. ¿Es la casa propia? | ☐ Sí ☐ No ☐ No contesta |
| 8-3. ¿Cuántos cuartos tiene su casa? | __________ |
| 8-4. ¿Cuántas personas duermen en su casa? | __________ |
| 8-5. ¿Cuántas personas trabajan para ganar ingreso monetario? |  |
| 8-6. ¿Tiene terreno o parcela para siembra? | ☐ Propia ☐ Alquilada  ☐ No tiene ☐ No contesta |
| 8-7. ¿Tiene electrodomésticos o aparatos eléctricos (refrigerador, televisión, o equipo de música) | ☐Sí ☐ No ☐ No contesta |
| 8-8. ¿Tiene acceso al agua para tomar en su casa? | ☐Sí ☐ No ☐ No contesta |
| 8-9. ¿Tiene letrina o inodoro? | ☐ Sí ☐ No ☐ No contesta |
| 8-10. ¿De dónde proviene el agua con que usted se baña? | ☐Ducha ☐ Almacenada en barril  ☐ Río ☐ Otros |
| 8-11. ¿Tiene algunos niños (6-15 años) que no asistan a la escuela? | ☐ Sí ☐ No ☐ No contesta |
| 8-12. ¿Si encuentra un chinche en su casa, que haría con él? | ☐ Entregarlo al Centro de Salud  ☐ Matarlo ☐ No haría nada  ☐ No sabe ☐ No contesta |

Ya hemos terminado, pero por favor deme unos minutos para revisar que hemos completado todas las preguntas. ¡Muchas Gracias por su participación!

Responsables de la encuesta (nombres, apellidos y firma)

Nosotros, como encuestadores, hemos completado este cuestionario debidamente:

1.

2.

Supervisor (nombres, apellidos y firma)

Yo, como supervisor, he revisado este cuestionario y aseguro que todas las preguntas fueron contestadas y los encuestadores anotaron las respuestas en la forma fácilmente entendible:

1.
