# Supplementary material for: The resilience of Triatoma dimidiata: An analysis of reinfestation in the Nicaraguan Chagas disease vector control program (2010–2016)
Source: PLoS One. 2018 Aug 23;13(8):e0202949. doi: 10.1371/journal.pone.0202949 (PMC6107243; doi:10.1371/journal.pone.0202949)
Supplement: S2 File — (DOCX) [file pone.0202949.s002.docx]

**INFORMED CONSENT FORM**

Dear community members, my name is ________________________________________________________

We are doing a survey to investigate the presence of *chinches*, transmitters of Chagas disease. The survey will be conducted by Nagasaki University, Japan, in collaboration with Ministry of Health in Nicaragua.

The information you give us is **confidential** and will not be shared with none that has no relation with this survey and will be utilized only for analyzing the risk of *chinches* in your community.

Your participation is voluntary, and you can withdraw from the survey at any moment or refuse answering any questions.

If you agree, I will ask you to give me your sign of consent, indicating that you agree to participate in this survey.

The consent form was read and explained to participant;

Signature of participant: _________________________________________________

Signature of investigator: _________________________________________________

Date: _________/___Feb__/ 2016

| Basic Information  1-1. Survey date: _/ Feb / 2016  1-2. House code:  1-3. Name of community/block:  1-4. Name of the head of family:  1-5. Is the name of the head of family the same as the one that appears in the survey list?  ☐ Yes ☐ No  1-6. How many years has the family lived in this house? ☐ More than 6 years ☐ Less than 5 years |
| --- |
| Coordinates (GPS) – UTM zona 16   \|  \|  \|  \|  \|  \|  \|  \| \| --- \| --- \| --- \| --- \| --- \| --- \| --- \| \|  \|  \|  \|  \|  \|  \|  \| |
| Predominant materials of the bedroom (investigator observation)  2-1. Wall: ☐ Bajareque without plaster ☐ Bajareque with plaster ☐ Adobe without plaster  ☐ Adobe with plaster ☐ Blocks/Bricks ☐ Concrete ☐ Wood  ☐ Prefabricated houses 　☐ Others:  2-2. Roof: ☐ Tin ☐ Tiles ☐ Straw/Palm leaves ☐ Others:  2-3. Floor: ☐ Dirt ☐ Concrete/Tiles ☐ Others: |
| Peridomestic conditions (Investigator observation)  3-1. Mark everything that you observe in peri-domestic areas (yard)  ☐ Stone fence ☐ Piled tiles ☐ Piled firewood  ☐ Henhouse ☐ Pig house ☐ Doghouse ☐ Bird cages  ☐ Other things where *chinches* can hide: |
| Results of Entomological Inspection  4-1. Time (only searching *chinches*, not including time to fill this questionnaire)  Starting time :  Finishing time :  4-2. Species of chinches captured:  ☐ *T. dimidiata* ☐ *R. prolixus* ☐ Others:  4-3. Number of chinches and other evidence captured:   \| Place \| Captured alive \| \| Other evidence \| \| \| \| --- \| --- \| --- \| --- \| --- \| --- \| \| Adults \| Nymphs \| Dead \| Shell \| Feces \| \| Intra-domestic \|  \|  \|  \|  \|  \| \| Peri-domestic \|  \|  \|  \|  \|  \| \| Total \|  \|  \|  \|  \|  \| |

Observations:

Questionnaire to the householder (or his/her equivalent)

| 5-1. How many animals do you have in your house?  [note numbers] | Dogs Cats  Pigs Birds  Chickens in henhouse  Chickens without henhouse |
| --- | --- |
| 5-2. Are there any animals that sleep within the house? | ☐ Yes ☐ No ☐ No response |
| 5-3. Have you seen rats or mice in your house? | ☐ Yes ☐ No ☐ No response |
|  |  |
| 6-1. Do you or your family members plaster the walls?  How frequent? | ☐ Yes ☐ No ☐ No response  every _______ days / weeks / months |
| 6-2. Do you or your family members spray your house?  How frequent? | ☐ Yes ☐ No ☐ No response  every _______ days / weeks / months |
|  |  |
| 7-1. (Show the photography) Do you know *chinches*? | ☐ Yes ☐ No ☐ No response |
| 7-2. Have you seen *chinches* in your house during last year? | ☐ Yes ☐ No ☐ No response |
| 7-3. Have you reported *chinches* to Health Centers during last year? | ☐ Yes ☐ No ☐ No response  *Jump to “8-1”* |
| 7-4 How many times have you reported *chinches* last year? | _______ times |
| 7-5. Have health personnel or volunteers visited your house after you reported *chinches*? | ☐ Yes ☐ No ☐ No response  *Jump to“8-1”* |
| 7-6. What kind of personnel visited you? | (Multiple responses)  ☐ Medical doctor ☐ Nurse  ☐ Vector control technicians ☐ Community volunteers  ☐ Others _____________________________ |
| 7-7. If the personnel or volunteer visited you after you reported *chinches*, what did he/she do in your house? | (Multiple response)  ☐ Gave a talk about *chinches*  ☐ Inspected where *chinches* were found  ☐ Sprayed the house  ☐ Others ____________________________ |
|  |  |
| 8-1. How many years ago did you constructed the house? | years |
| 8-2. Is your own house? | ☐ Yes ☐ No ☐ No response |
| 8-3. How many rooms are there in the house? | __________ |
| 8-4. How many persons sleep in the house? | __________ |
| 8-5. How many persons work to generate monetary income? |  |
| 8-6. Do you have fields for agriculture? | ☐ Owner ☐ Rent  ☐ No ☐ No response |
| 8-7. Do you have electric home appliance (refrigerator, television, or music equipment) | ☐ Yes ☐ No ☐ No response |
| 8-8. Do you have access to water to drink in your house? | ☐ Yes ☐ No ☐ No response |
| 8-9. Do you have latrine or toilet? | ☐ Yes ☐ No ☐ No response |
| 8-10. Where does the water for bathing come from? | ☐ Shower ☐ Stored in drums  ☐ River ☐ Others |
| 8-11. Do you have any children (6-15 years old) who do not go to the school? | ☐ Yes ☐ No ☐ No response |
| 8-12. If you find *chinches* in your house, what do you do with them? | ☐ Report to Health Center  ☐ Kill ☐ Do nothing  ☐ I don’t know ☐ No response |

Now we finished, but please give me a few minutes to check if we completed all questions. Thank you for your participation.

Persons in charge of the survey (first names, last names and sign)

We, as investigators, have completed this questionnaire accordingly:

1.

2.

Supervisor (first name, last name and sign)

I, as supervisor, have reviewed this questionnaire and confirm that all questions were addressed and the investigators noted the responses accordingly:

1.
